# Community-integrated omics links dominance of a microbial generalist to fine-tuned resource usage

**DOI:** 10.1038/ncomms6603

**Published:** 2014-11-26

**Authors:** Emilie E. L. Muller, Nicolás Pinel, Cédric C. Laczny, Michael R. Hoopmann, Shaman Narayanasamy, Laura A. Lebrun, Hugo Roume, Jake Lin, Patrick May, Nathan D. Hicks, Anna Heintz-Buschart, Linda Wampach, Cindy M. Liu, Lance B. Price, John D. Gillece, Cédric Guignard, James M. Schupp, Nikos Vlassis, Nitin S. Baliga, Robert L. Moritz, Paul S. Keim, Paul Wilmes

**Affiliations:** 1 Luxembourg Centre for Systems Biomedicine, University of Luxembourg, 7 Avenue des Hauts-Fourneaux, L-4362 Esch-sur-Alzette, Luxembourg; 2 Institute for Systems Biology, 401 Terry Avenue North, Seattle, Washington 98109, USA; 3 TGen North, 3051 West Shamrell Boulevard, Flagstaff, Arizona 86001, USA; 4 Centre de Recherche Public-Gabriel Lippmann, 41 rue du Brill, L-4422 Belvaux, Luxembourg

## Abstract

Microbial communities are complex and dynamic systems that are primarily structured according to their members’ ecological niches. To investigate how niche breadth (generalist versus specialist lifestyle strategies) relates to ecological success, we develop and apply an integrative workflow for the multi-omic analysis of oleaginous mixed microbial communities from a biological wastewater treatment plant. Time- and space-resolved coupled metabolomic and taxonomic analyses demonstrate that the community-wide lipid accumulation phenotype is associated with the dominance of the generalist bacterium *Candidatus* Microthrix spp. By integrating population-level genomic reconstructions (reflecting fundamental niches) with transcriptomic and proteomic data (realised niches), we identify finely tuned gene expression governing resource usage by *Candidatus* Microthrix parvicella over time. Moreover, our results indicate that the fluctuating environmental conditions constrain the accumulation of genetic variation in *Candidatus* Microthrix parvicella likely due to fitness trade-offs. Based on our observations, niche breadth has to be considered as an important factor for understanding the evolutionary processes governing (microbial) population sizes and structures *in situ*.

## Abstract (web summary)

Within microbial communities, microorganisms adopt different lifestyle strategies to use the available resources. Here, the authors use an integrated ‘multi-omic’ approach to study niche breadth (generalist versus specialist lifestyles) in oleaginous microbial assemblages from an anoxic wastewater treatment tank.

## Graphical abstract

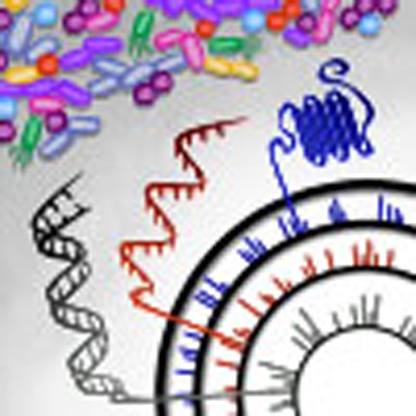

Microorganisms are ubiquitous and form complex, heterogeneous and dynamic assemblages^1^. They represent essential components of the Earth’s biogeochemical cycles^2^, human metabolism^3^ and biotechnological processes^4^. Microbial population sizes and structures are governed by resource availability and usage^5^,^6^,^7^, and mainly develop in response to the breadths of the fundamental and realized niches of constituent populations^8^,^9^ (narrow for specialists; wide for generalists) albeit being influenced by stochastic neutral processes^8^,^9^. Microbial niche breadths remain poorly described *in situ* (for an earlier study, see ref. 10). The application of high-fidelity, high-resolution and high-throughput molecular analyses to microbial consortia holds great promise for resolving population-level phenotypes and defining their corresponding niche breadths *in situ*^11^. However, to obtain community-wide multi-omic data that can be meaningfully integrated and analysed, systematic measurements are essential^1^.

We have recently developed the required laboratory methods that enable us to isolate representative biomolecular fractions (DNA, RNA, proteins and small molecules) from single microbial community samples^12^,^13^. Here, we expand this concept by performing integrated omic analyses of purified biomolecular fractions from oleaginous mixed microbial communities (OMMCs) located on the surface of an anoxic biological wastewater treatment tank to study how microbial lifestyle strategies relate to ecological success and the associated community-level phenotypes in this fluctuating but well-characterized environment. In addition, OMMCs are typically enriched in lipid-accumulating filamentous bacteria and often associated with operational difficulties, such as phase separation and bulking in biological wastewater treatment plants^14^. However, the phenotypic traits of OMMCs may allow for the recovery of lipids from wastewater streams for subsequent chemical energy recovery through biodiesel synthesis^15^. As for other microbial consortia, a detailed understanding of OMMC ecology is essential for the formulation of strategies to shape microbial community structure and function (in this case, enriching for lipid-accumulating bacteria) in the future. Building on the recently developed methodologies for the systematic molecular characterization of microbial consortia^12^,^13^, and for resolving and reconstructing population-level genomic complements from community-wide sequence data^16^, here, we integrate multi-omic data sets to resolve microbial lifestyle strategies *in situ*, identify finely tuned gene expression governing resource usage by a dominant bacterial generalist population and reveal that genetic variation within this population is constrained likely due to fitness trade-offs.

## Results

### Coupled metabolomic—taxonomic analyses over space and time

**Figure 1:**
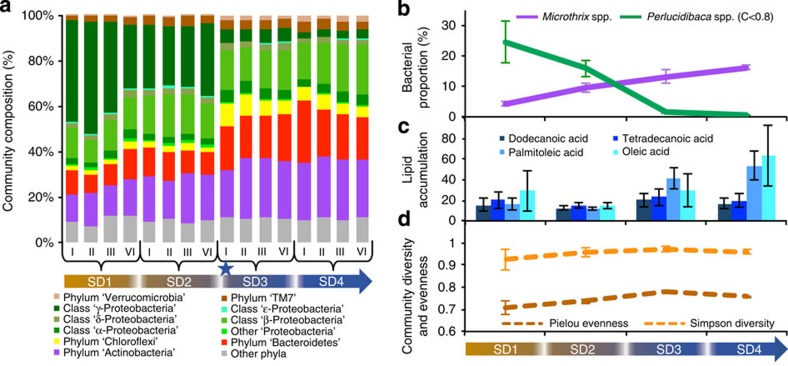
Microbial community dynamics and lipid accumulation from wastewater. (**a**) Fractions of taxa identified across the communities sampled on four distinct dates (SD1–SD4). Roman numerals refer to the four biological replicates sampled per time point. The blue star indicates the representative sample from SD3 subjected to the integrated omic analysis. (**b**) Average genus-level abundances of the two dominant populations. The most abundant microbial population in winter was identified as *Candidatus* Microthrix spp., whereas a population tentatively identified (confidence level <0.8) as *Perlucidibaca* spp. was dominant in autumn. (**c**) Long-chain fatty acid intracellular accumulation per sampling date expressed as ratios between quantified intracellular and extracellular long-chain fatty acid abundances. (**d**) Genus-level alpha diversity and evenness. (**b**–**d**), error bars represent s.d. (*n*=4).

To obtain a detailed view of OMMC (Supplementary Fig. 1a) lipid accumulation and bacterial composition, we first applied coupled metabolomics and 16S rRNA gene sequencing to samples taken over space and time (see the Methods section). The initial sample set included four distinct biological replicates (Supplementary Fig. 1b) from four representative time points (two in autumn, two in winter; Methods and Supplementary Fig. 2a,b). Using gas chromatography coupled to tandem mass spectrometry (GC-MS/MS), absolute quantifications of the 14 major long-chain fatty acids were obtained for OMMC biomass and wastewater, respectively (Methods). The V3 and V6 hypervariable regions of the 16S rRNA gene were amplified from the DNA fraction of the samples (Methods). The barcoded amplicons were pyrosequenced on a 454 GS FLX platform, yielding a total of 265,592 reads (*n*=10,574±3,451 (mean±s.d.) per OMMC sample after quality control and chimera filtering). Direct taxonomic classification of the obtained sequencing reads demonstrates that, at the phylum level, the OMMCs of the studied treatment plant were dominated across the studied seasons by Proteobacteria and Actinobacteria, which constituted 43%±14% and 21%±5% (mean±s.d.) of the community, respectively (Fig. 1a). Similar results were obtained when operational taxonomic unit clustering was applied before classification (Supplementary Fig. 3a). Among the two most dominating taxa over time (Fig. 1b; Supplementary Fig. 3b), *Candidatus* Microthrix spp., a well-known lipid-accumulating genus^17^,^18^, correlated with a more pronounced community-wide lipid accumulation phenotype (Spearman correlation coefficient *ρ*≥0.8 for *Candidatus* Microthrix spp. and palmitoleic and oleic acids, respectively; Fig. 1c). This trend, despite the metabolic versatility of *Candidatus* Microthrix spp.^17^,^18^ and the statistically significant lower levels of lipids available compared with other carbon and energy sources particularly in winter (Wilcoxon rank-sum test, *P*<0.001, *n*=15; Supplementary Fig. 4), suggests optimal foraging behaviour^19^,^20^ by *Candidatus* Microthrix spp.

### High-throughput multi-omic analyses

To obtain an initial view of the population-level characteristics that determine the ecological success of *Candidatus* Microthrix spp. in winter in comparison with other co-occurring microbial populations, we first conducted a detailed integrated omic analysis of a single representative sample obtained on sampling date 3 (SD3; Methods). This sample was selected on the basis of the relatively large *Candidatus* Microthrix spp. population size (Fig. 1b), the desired community-wide lipid accumulation phenotype (Fig. 1c) and its even but diverse community composition (Fig. 1d). Concomitantly isolated DNA, RNA and protein fractions were processed and subjected to high-throughput metagenomic, metatranscriptomic and metaproteomic analyses. Massive parallel sequencing of DNA and cDNA resulted in the generation of 1.47 × 10^7^ and 1.65 × 10^7^ metagenomic and metatranscriptomic paired-end reads, respectively. Shotgun proteomic analyses based on liquid chromatography followed by tandem mass spectrometry (LC-MS/MS) resulted in the generation of 271,915 mass spectra (Methods).

### Assembly-free community profiling

To assess the composition of the winter OMMC, we first carried out an assembly-free community analysis. For this, the results obtained using two shotgun sequence data profiling tools, namely MetaPhlAn^21^ and MG-RAST^22^, were compared with the profiles obtained using the previous 16S rRNA gene sequencing. Given the poor taxonomic classification of *Candidatus* Microthrix spp. in the databases used by these profiling tools, all analyses were limited to phylum-level classification. At this level, similar community structures were apparent for the representative sample from SD3 using the three distinct approaches (Supplementary Fig. 3a and Methods).

Second, to resolve the functions encoded and expressed by the OMMC from SD3, the proportions of genes and transcripts belonging to different cluster of orthologous group (COG) functional categories were compared for both the metagenomic and metatranscriptomic data sets (Supplementary Fig. 5). Similar proportions were observed for most of the different functional categories in both data sets. Nevertheless, major differences were observed for the categories ‘J—translation, ribosomal structure and biogenesis’, ‘O—posttranslational modification, protein turnover, chaperones’, and ‘C—energy production and conversion’. Differences in gene copy numbers and transcript abundances may be expected for these functional genes because of their typical high levels of constitutive expression. The proportion of gene copies and transcript numbers were similar for the COG category ‘I—lipid transport and metabolism’ although these genes are expected to have essential roles in OMMCs and, therefore, overall high levels of expression may be expected. The previous findings suggest that key members in the OMMC, that is, *Candidatus* Microthrix spp., are involved in lipid transformations. Consequently, key processes related to lipid transport and metabolism, that is, resource usage, have to be resolved at the population level. Therefore, to deconvolute the activities of the constituent OMMC members, a detailed population-resolved analysis was subsequently performed.

### Population-resolved integrated omic analyses

**Figure 2:**
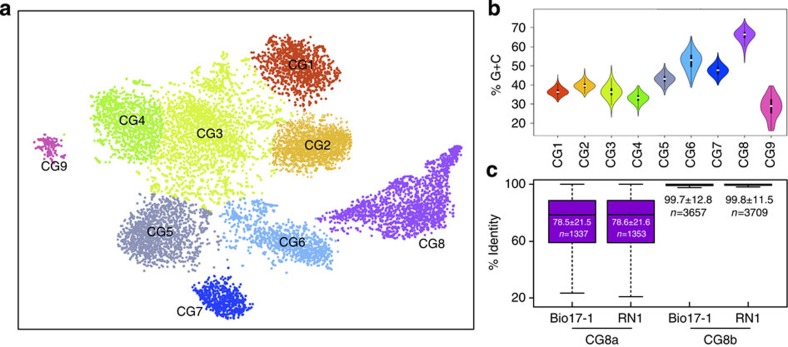
Identification of genomic fragments derived from distinct populations. (**a**) Binning of assembled contigs (≥1,000 bp in length) on the basis of pentanucleotide signatures and visualization using the BH-SNE algorithm followed by human-augmented clustering of composite genome (CG) groups. (**b**) Violin plots of the G+C percentage for contigs within each of the CG groups. (**c**) Percentage amino-acid identity of the two subpopulations in CG8 (CG8a and CG8b) compared with the two sequenced *Candidatus* Microthrix parvicella (Bio17-1 (ref. 16) and RN1 (ref. 17)) genomes. The values are median±s.d. and *n* is the number of putative orthologues identified as best BLAST hits. Boxplots represent the lower quartile, median and upper quartile. Whiskers are placed at × 1.5 interquartile range beyond the lower and upper quartiles.

To resolve the traits of the dominant populations within the community obtained on SD3, composite genomes (CGs) were reconstructed using a newly developed iterative binning and *de novo* assembly procedure for the combined metagenomic and metatranscriptomic sequence data (Methods). Detailed profiling and grouping of the assembled contiguous sequence fragments (contigs) were performed on the basis of centred log-ratio transformed pentanucleotide signatures and visualization using the Barnes–Hut Stochastic Neighbour Embedding (BH-SNE) algorithm^16^, followed by human-augmented clustering (Methods). Using this approach, we identified nine CG groups (Fig. 2a) that displayed homogeneous G+C percentage (Fig. 2b). The assembled contigs from the nine CG groups were subjected to gene calling and annotation (Methods), which led to the identification of 23,317 coding sequences, with 16,841 and 1,533 of these being represented at the transcript and protein levels, respectively.

The refinement of the CGs by depth of read coverage resulted in the splitting of CG8 into low (CG8a) and high (CG8b) coverage populations (Supplementary Fig. 6). The average amino-acid sequence identities of CG8b with recently obtained genome sequences of *Candidatus* Microthrix parvicella strains Bio17-1 (ref. 17) and RN1 (ref. 18) were >99% (Fig. 2c), an identity level usually observed among strains of the same bacterial species^23^. In contrast, the CG8a identity levels compared with the same reference sequences were around 78% (Fig. 2c). Consequently, CG8b represents a *Candidatus* Microthrix parvicella population in this OMMC sample from SD3.

The identities of the other CGs were determined using 31 phylogenetic marker genes^24^ resulting, for example, in the tentative identification of CG5 as a population belonging to the Moraxellaceae family of the γ-Proteobacteria (Supplementary Data 1).

Eight of the 10 reconstructed CGs were estimated to be >60% complete with CG8b, CG4 and CG5 being 97.5, 90 and 85% complete, respectively (Supplementary Data 1). On the basis of population sizes inferred from the mapping of the metagenomic read data onto the CGs (Methods), these three community members represent the first, the seventh and the fourth most abundant OMMC populations, respectively. Because CG8b and CG5 represent the most deeply covered nearly complete genomic reconstructions, a detailed analysis of the ecophysiology of these populations based on the generated functional omic data was performed.

**Figure 3:**
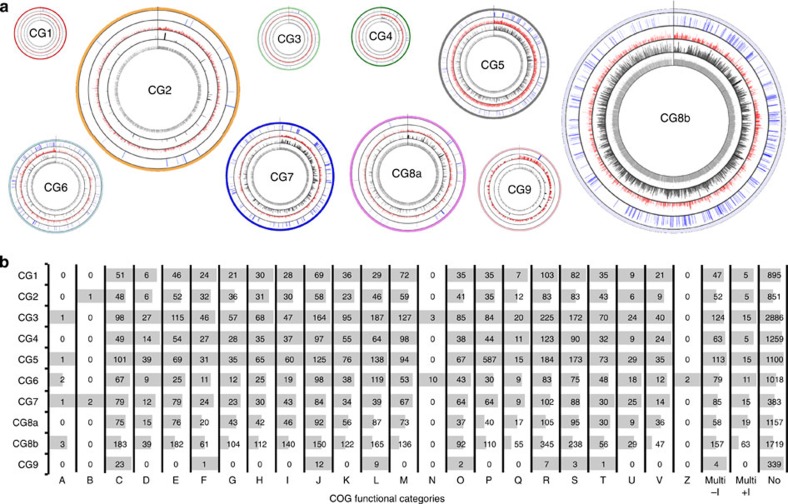
Population-resolved integrated omics. (**a**) Circos plots^63^ of genome-wide gene expression levels for the 10 reconstructed composite genomes (CGs). Tracks (from the innermost concentric track to the outermost): log_10_ of metagenomic fragments per 1 kb of sequence per 10^6^ mapped reads (FPKM^46^; dark grey), log_10_ of the numbers of detected SNPs per gene (black), log_10_ of metatranscriptomic FPKM (red), log_2_ of the protein expression levels as Normalized Spectral Indices (NSI; blue), and reconstructed contigs ordered by size. The track scales are identical across the plots. The sizes of the individual Circos plots are weighted according to the log_10_ of inferred population size (Methods). (**b**) The number of genes for each COG category encoded by the different CGs and their corresponding relative transcript levels (grey bars). COG categories are: A, RNA processing and modification; B, chromatin structure and dynamics; C, energy production and conversion; D, cell cycle control, cell division, chromosome partitioning; E, amino acid transport and metabolism; F, nucleotide transport and metabolism; G, carbohydrate transport and metabolism; H, coenzyme transport and metabolism; I, lipid transport and metabolism; J, translation, ribosomal structure and biogenesis; K, transcription; L, replication, recombination and repair; M, cell wall/membrane/envelope biogenesis; N, cell motility; O, posttranslational modification, protein turnover, chaperones; P, inorganic ion transport and metabolism; Q, secondary metabolites biosynthesis, transport and catabolism; R, general function prediction only; S, function unknown; T, signal transduction mechanism; U, intracellular trafficking, secretion and vesicular transport; V, defence mechanisms; Z, cytoskeleton; Multi−I, multiple COG category excluding I; Multi+I, multiple COG category including I; No, no COG category assigned.

By mapping the metatranscriptomic and metaproteomic data onto the reconstructed CGs (Methods), all 10 populations were found to be active albeit exhibiting differing levels of gene expression (Fig. 3a, Supplementary Figs 7–9, Supplementary Data 2). Observed patterns of gene expression were not necessarily consistent at the transcript and protein levels for the different CGs. Discrepancies between the levels of gene expression inferred from transcriptomic and proteomic data have been well described in eukaryotes^25^ and these have also recently been observed for microbial communities^26^. The lack of correlation may have different reasons including differing molecular half-lives^26^ and/or possible posttranscriptional or posttranslational modifications, which are not detectable using the transcriptomic and proteomic methodologies used in this study.

Despite its large population size, population CG8b expressed only a comparatively small fraction (45.8% of possible transcripts detected) of its genetic complement *in situ* (Fig. 3b, Supplementary Data 1, Supplementary Fig. 8) and this at a moderate level of expression both at the transcript and protein levels (Supplementary Fig. 9a), suggesting the fine-tuning of gene expression by CG8b. On the contrary, the other CGs exhibited expression of the majority of their genetic repertoire (Fig. 3, Supplementary Data 1, Supplementary Fig. 8). In particular, 92.7% of CG5 genes were detected at the transcript level.

**Figure 4:**
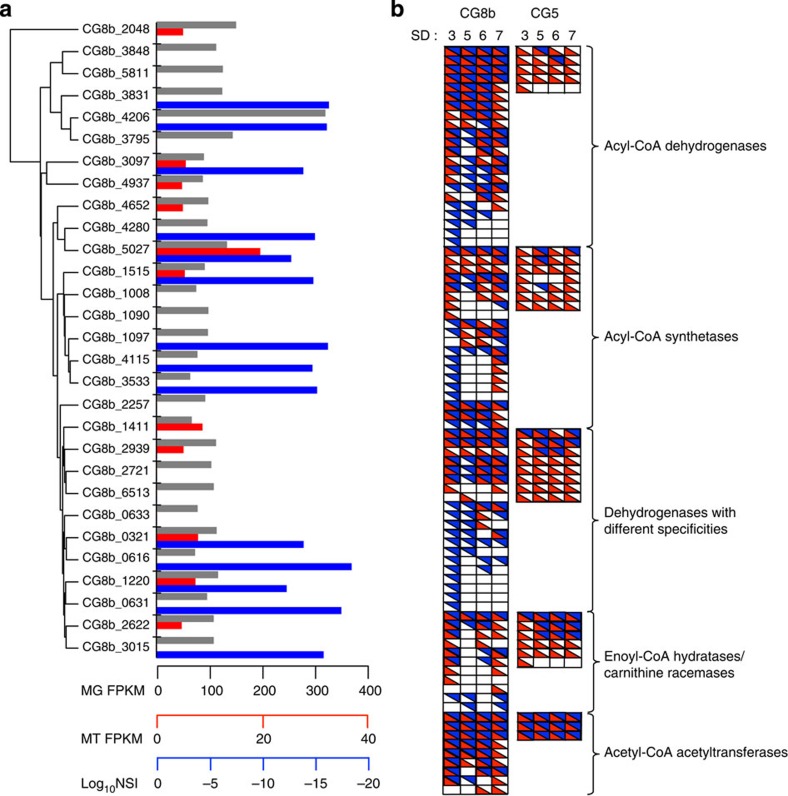
*In situ* fine-tuning of gene expression by *Candidatus* Microthrix parvicella. (**a**) Dendrogram based on the amino acid similarities of 29 predicted long-chain fatty acid-CoA ligases encoded by the *Candidatus* Microthrix parvicella population (CG8b). Metagenomic (grey) and metatranscriptomic (red) data represented as fragments per 1 kb of sequence per 10^6^ mapped reads (FPKM), the protein abundance data (blue) is represented as the log_10_ of the Normalized Spectral Indices (NSI). (**b**) Qualitative gene expression patterns of the five most prevalent homologous gene sets belonging to the COG category ‘I—lipid transport and metabolism’ encoded by *Candidatus* Microthrix parvicella (CG8b) and the CG5 population at the metatranscriptomic (red) and metaproteomic (blue) levels across four sampling time points.

On the basis of its genetic repertoire, *Candidatus* Microthrix parvicella appears to be physiologically versatile^17^,^18^ which, combined with its enrichment under fluctuating environmental conditions, indicates a generalist lifestyle strategy^27^. The fine-tuning of gene expression is particularly apparent for genes involved in lipid transport and metabolism, which show a clear genomic enrichment within the CG8b population although only a limited subset, that is, 46%, are expressed (Fig. 3b, Supplementary Fig. 8). Among these genes, long-chain fatty acid-CoA ligases are essential for the assimilation and activation of extracellular fatty acids into their acyl-CoA conjugates^28^ and are therefore required for resource usage by *Candidatus* Microthrix parvicella. CG8b encodes 29 genes annotated as homologues of this enzyme class, indicating that a broad spectrum of free fatty acids may be assimilated by this population. Only 11 and 14 of these genes were found to be expressed at the RNA and protein levels, respectively (Fig. 4a). In contrast, the five genes annotated as long-chain fatty acid-CoA ligase homologues in CG5 were all expressed (Supplementary Data 2). This observation suggests the fine-tuned expression of these genes by *Candidatus* Microthrix parvicella, likely through the tight regulation of gene expression, to facilitate preferential resource usage in accordance with optimal foraging behaviour^19^,^20^.

### Population-level genetic diversity

To assess the overall frequencies of population-level genetic variation and determine how these variations may reflect the lifestyle strategy of CG8b, the number of single-nucleotide polymorphisms (SNPs) identified in individual CGs were normalized according to reconstructed CG length and population sizes inferred from the proportion of metagenomic reads mapped to the reconstructed CGs (Supplementary Data 1). CG8b displayed a relatively limited level of genetic variation. For example, it exhibited one order of magnitude fewer SNPs compared with CG5, the other almost complete reconstructed CG with enough coverage to confidently infer SNP densities (Supplementary Data 1). Given the generalist lifestyle strategy of CG8b, the relatively high within-population genetic homogeneity may be explained by fitness trade-offs resulting, for example, from antagonistic pleiotropy^29^,^30^. In a fluctuating environment, most of the beneficial or neutral mutations under one condition may be deleterious under other conditions, thereby restricting the evolutionary rate of generalists^29^,^30^. An alternative hypothesis positing that this low population-level variation may be due to a recent genetic bottleneck (selective sweep, colonization, and so on.) followed by population expansion^31^ may be rejected on the basis of the high genetic similarity between the reconstructed CG and the two available *Candidatus* Microthrix parvicella genome sequences from strains isolated from distant wastewater treatment plants 7 and 16 years before the present study (Fig. 2c).

### Fine-tuned gene expression and limited genetic diversity over time

To validate the previous snapshot views of the ecophysiology and structure of populations CG8b and CG5, identical multi-omic analyses were carried out on three additional, rationally selected OMMC samples from the same wastewater treatment tank (Methods). A first additional sample was collected on SD7 approximately a year after SD3 when the measured physico-chemical parameters were very similar to those measured on SD3 (Supplementary Fig. 2b,c). In addition, samples were selected from SD5 and SD6 because the physico-chemical parameters on these dates were at variance with SD3 and SD7 (Supplementary Fig. 2c). Importantly, the additional samples also contain populations CG8b and CG5 at sufficient quantities to obtain the necessary coverages at the genomic and transcriptomic levels for the subsequent analyses of genetic diversity and gene expression over time (Table 1). Massive parallel sequencing of DNA and cDNA resulted in the generation of an additional 5 × 10^7^ and 4.45 × 10^7^ metagenomic and metatranscriptomic paired-end reads, respectively. In addition, a total of 326,630 additional mass spectra were generated using LC-MS/MS (Methods).

To corroborate the fine-tuning of gene expression of the generalist population CG8b (*Candidatus* Microthrix parvicella) deduced from the analysis of the sample from SD3, patterns of gene expression were assessed for SD5–SD7. Although the CG5 population consistently expressed the vast majority of its genetic repertoire, only a comparatively small fraction of the genetic complement of CG8b was expressed at each additional time point despite its relatively consistent population size (Table 1, Supplementary Fig. 9b, Supplementary Data 3). These observations support the previous results obtained on the OMMC from SD3. In addition, analogous to the patterns observed for SD3, the expression of genes involved in lipid transport and processing encoded by CG8b was highly variable over time (Fig. 4b). In contrast, the CG5 population consistently expressed the vast majority of this functional category (Fig. 4b, Supplementary Fig. 10). These comprehensive additional data reinforce the notion of finely tuned gene expression for resource usage by the *Candidatus* Microthrix parvicella generalist population.

The patterns of low SNP density in the generalist population CG8b were also consistent over time, with one order of magnitude fewer SNPs generally apparent in CG8b compared with CG5 (Table 1, Supplementary Data 4). In contrast to CG5, the variant counts of CG8b remain relatively constant over time (Table 1). The observations from the three additional time points reinforce the previous notion that a generalist lifestyle under fluctuating environmental conditions constrains the accumulation of population-level genetic variation.

## Discussion

Here, the application of systematic integrated multi-omic measurements to mixed microbial communities has allowed us to obtain fundamental insights into the ecology of the constituent dominant populations. On the basis of its genetic repertoire and enrichment under temporally changing environmental conditions, the dominant population within the winter OMMCs, that is, *Candidatus* Microthrix parvicella, can be classified as a generalist species. The low proportion of genes expressed over time indicates that its ecological success most likely results from finely tuned gene expression facilitating optimal foraging behaviour. In addition, the *Candidatus* Microthrix parvicella population exhibits low levels of genetic variation that may be explained by evolutionary constraints resulting from fitness trade-offs. Elucidating the exact mechanisms driving these trade-offs in *Candidatus* Microthrix parvicella, for example, antagonistic pleiotropy or others, will require additional integrated omic data sets to be generated from many more samples taken over space and time, as well as surveys of other wastewater treatment plants. Overall, our results call for similar studies on other microbial communities to determine whether fine-tuning of gene expression is a general feature of generalists and whether lifestyle strategies provide an explanation for the varying degrees of within-population genetic heterogeneity so far observed in metagenomic data sets^32^.

## Methods

### Sample processing

Oleaginous mixed microbial communities (OMMCs) were sampled at four representative time points from the surface of the anoxic treatment phase of a biological wastewater treatment plant treating residential effluents (Schifflange, Esch-sur-Alzette, Luxembourg; 49°30′48.29′′N; 6°1′4.53′′E). For each sampling date (SD), four distinct ‘islets’ were collected (herein referred to as biological replicates; Supplementary Fig. 1), transferred into a sterile tube, snap frozen on site and maintained at −80 °C until processing. Initial samples were taken on 4 October 2010 (SD1; anoxic tank wastewater temperature of 20.7  °C), 25 October 2010 (SD2; 18.9 °C), 25 January 2011 (SD3; 14.5 °C) and 23 February 2011 (SD4; 13.9 °C). Since the prevalence of OMMCs is dependent on wastewater temperature^14^, these samples were chosen to be representative of the range of wastewater temperatures at which OMMCs are highly abundant within the system. Due to heavy precipitation, which leads to dispersion of the OMMC islets, and due to excess nitrate concentrations (Supplementary Fig. 2a), no samples from December/early January were included in the present study. However, given the range of water temperatures encountered in this biological wastewater treatment plant (Supplementary Fig. 2a), the October as well as January and February samples are representative of autumn and winter OMMCs, respectively.

For the integrated omic analyses of a representative sample, a single biological replicate (SD3-I; Fig. 1a) from the 25 January 2011 samples was selected for subsequent high-resolution omic analyses. This sample was chosen on the basis of its pronounced community-wide lipid accumulation phenotype, dominance of *Candidatus* Microthrix spp., and because it exhibited the highest bacterial diversity and evenness. This in turn should allow a comprehensive community-wide overview and reconstruction of composite genomes (CGs) from the most abundant populations within the OMMC.

To validate findings from the integrated omic analysis of the sample from SD3, three additional OMMC samples were rationally selected. On the basis of hierarchical clustering analysis of physico-chemical parameters (Supplementary Fig. 2c), a first additional sample was collected on SD7 (11 January 2012) approximately a year after SD3 when the measured physico-chemical parameters were very similar to those for SD3 (Supplementary Fig. 2b,c). In addition, samples were selected from SD5 (5 October 2011) and SD6 (12 October 2011) because the physico-chemical parameters measured on these dates (especially wastewater temperature) were at variance with those of SD3 and SD7 (Supplementary Fig. 2b,c).

### Biomolecular isolation

All biomolecular fractions were obtained using a recently developed methodological framework, which allows recovery of high-quality biomolecular fractions (DNA, RNA, proteins, polar and non-polar metabolites) from unique undivided samples^12^,^13^. For biomacromolecular purification we used the AllPrep DNA/RNA/Protein Mini kit (Qiagen). Resulting biomolecular fractions comprising genomic DNA, RNA, proteins and small molecules were further processed and analysed as detailed below.

### Quantification of biomolecular resources

Intracellular and extracellular non-polar metabolite fractions of the four biological replicates collected on SD1 to SD4 were obtained using the biomolecular extraction procedure described earlier^12^,^13^ (only on three biological replicates for SD3). The non-polar phases were aliquoted in four vials (analytical replicates) of 100 μl each, dried overnight and the resulting pellets were then preserved at −80 °C. The intracellular and extracellular dried extracts were redissolved in 100 and 40 μl of dichloromethane, respectively. Derivatisation was carried out on 40 μl of solubilized extract with 40 μl of N,O-bis(trimethylsilyl)trifluoroacetamide:trimethylchlorosilane 99:1 (Sylon BFT, Supelco) for 1 h at 70 °C. Samples were analysed by gas chromatography coupled to tandem mass spectrometry (GC-MS/MS) on a Thermo Trace GC and a Thermo TSQ Quantum XLS triple-quadrupole MS (Thermo Fisher). Samples were injected in PTV splitless mode onto a Rxi-5Sil MS column (20 m × 0.18 mm × 0.18 μm, Restek). Helium was used as the carrier gas at a constant flow rate of 1.0 ml min^−1^. Metabolite detection was performed in Multiple Reaction Monitoring mode, with two Multiple Reaction Monitoring transitions per target compound. Quantification was carried out by external calibration using standard mixtures of pure hexanoic acid, octanoic acid, decanoic acid, dodecanoic acid, tetradecanoic acid, palmitoleic acid, hexadecanoic acid, linoleic acid, oleic acid, linolenic acid, octadecanoic acid, eicosanoic acid, docosanoic acid and tetracosanoic acid, respectively (Sigma-Aldrich).

Total carbohydrate and protein quantities were determined on supernatant of the same samples comprising 200 mg of OMMC biomass for each sampling date using a Total Carbohydrate Assay Kit and a Total Protein Assay Kit (Micro Lowry, Peterson’s Modification; Sigma-Aldrich) according to the manufacturer’s instructions.

### 16S rRNA amplicon sequencing and analysis

*Amplification*. The wet-laboratory and bioinformatic procedures for analysing the bacterial community composition based on the V3–V6 region of the bacterial 16S rRNA gene are described in detail elsewhere^33^. Briefly, we generated barcoded V3–V6 amplicons using broad-coverage fusion PCR primers (forward primer: 5**′**- CCATCTCATCCCTGCGTGTCTCCGACTCAG*nnnnnnnn***CCTACGGGDGGCWGCA** -3′ and reverse primer 5′- CCTATCCCCTGTGTGCCTTGGCAGTCTCAG**CTGACGACRRCCRTGCA** -3′ with the underlined portion denoting FLX Lib-L adaptor sequences, italicized portion denoting the sample-specific 8-nt barcode sequences and bold portion denoting 16S rRNA gene primer sequences) on 10 μl of DNA extracts in 50 μl PCR reactions. The barcoded amplicons were pooled and sequenced on a Roche/454 Genome Sequencer FLX platform (Roche Applied Sciences). Resulting pyrosequencing data underwent processing and stringent filtering, which included chimera-checking, demultiplexing and quality-based trimming.

*Direct classification*. The processed pyrosequences were classified at each taxonomic level using a bootstrap confidence level of ≥80 and using a re-trained version of the Ribosomal Database Project (RDP) Naive Bayesian Classifier 2.4 (refs 34, 35), which includes the genus *Candidatus* Microthrix as a separate taxon. The training set consisted of the RDP 16S rRNA training set #9, with sequences S001942289, S000724117, S000724133, S001448117, S001448118, S001942070, S001942206, S002416756, S002416776, S000014283, S000588187, S000588192, S000010408, S000011228, S000021841, S000267158, S000588182, S000588183, S000588184, S000588185, S000588186, S000588188, S000588189, S000588190, S000588191, S000588193, S000724113, S000724122, S000832952, S000935760, S001294363, S001942173 reclassified as bacteria>Actinobacteria> Actinobacteria>unclassified>unclassified *Candidatus* Microthrix, by placing the full 16S rRNA gene sequence of the recently sequenced strain Bio17-1 (ref. 17) into the same taxon. Classification results from each sample were used to produce an abundance matrix for data analysis.

The 16S rRNA gene-based data of the four different biological replicates (islets) per sampling date were used for calculating Simpson diversity and Pielou evenness indices with 10 replications of subsampling of 6,359 reads per sample using the R Vegan package.

*Operational taxonomic unit-based classification*. In addition to the direct classification, processed pyrosequences were also analysed by clustering the reads into operational taxonomic units using Mothur^36^ v.1.32.1. To allow appropriate sample-specific classifications, the *Candidatus* Microthrix parvicella Bio17-1 (ref. 17) 16S rRNA gene sequence was added to the Mothur-formatted version of the RDP training set v9 and the related taxonomy file. Operational taxonomic units clustering was performed at a cut-off level of 0.03 before the assignment of taxonomy. Scripts are available from the authors upon request.

### Metagenome and metatranscriptome sequencing and assembly

*DNA library preparation*. The purified DNA fractions^12^,^13^ from the unique biological replicates I of SD3 and from SD5 to SD7 suspended in elution buffer (pH 8.0) were used to prepare a paired-end library with the AMPure XP/Size Select Buffer Protocol as previously described by Kozarewa *et al*.^37^, modified to allow for size selection of fragments using the double solid phase reversible immobilization procedure described earlier^38^. Size selection yielded metagenomic library fragments with a mean size of 450 bp. All enzymatic steps in the protocol were performed using the Kapa Library Preparation Kit (Kapa Biosystems) with the addition of 1 M PCR-grade betaine in the PCR reaction to aid in the amplification of high G+C percentage content templates.

*RNA library preparation*. Following RNA purification^12^,^13^ from the unique biological replicates I of SD3 and from SD5 to SD7, RNA fractions were ethanol precipitated, overlayed with RNAlater solution (Ambion) and stored at −80 °C. Before sequencing library preparation, the RNA pellet was rinsed twice in 80% ethanol and twice in 100% ethanol to remove any excess RNAlater solution. The pellet was then left on ice to dry. After ethanol evaporation, the RNA pellets were resuspended in 1 mM sodium citrate buffer at pH 6.4. Ribosomal RNAs were depleted using the Ribo-Zero Meta-Bacteria rRNA Removal Kit (Epicentre) according to the manufacturer's instructions. Transcriptome libraries were subsequently prepared using the ScriptSeq v2 RNA-Seq Library Preparation Kit (Epicentre) according to the manufacturer’s instructions. The resulting cDNA was subjected to Illumina sequencing.

*Nucleic acid sequencing*. Nucleic acid fractions were sequenced on an Illumina Genome Analyser (GA) IIx sequencer. Massive parallel sequencing of DNA and cDNA resulted in the generation of 1.47 × 10^7^ and 1.65 × 10^7^ metagenomic and metatranscriptomic paired-end reads for SD3, respectively. Similarly, the sequencing of SD5-derived DNA and cDNA generated 1.57 × 10^7^ and 1.47 × 10^7^ metagenomic and metatranscriptomic paired-end reads, SD6-derived DNA and cDNA sequencing generated 1.47 × 10^7^ and 1.48 × 10^7^ metagenomic and metatranscriptomic paired-end reads and SD7-derived DNA and cDNA sequencing generated 1.96 × 10^7^ and 1.80 × 10^7^ metagenomic and metatranscriptomic paired-end reads.

*Nucleic acid sequence data analysis*. MetaPhlAn^21^ (using default parameters) was used on 5' seven base pairs hard-clipped raw paired-end reads, collapsed, filtered at or above a mean QV of 30 and a minimum length of 60 bp.

Raw metagenomic paired-end reads were submitted to MG-RAST^22^ using the ‘join fastq-formatted paired-reads’ option retaining the non-overlapping reads, dynamic trimming and dereplication options. Raw metatranscriptomic reads were submitted to MG-RAST as described for metagenomic data, except that the dereplication option was not selected. As MG-RAST also supports the analysis of eukaryotic sequences, to allow comparison to MetaPhlAn and the 16S rRNA gene sequencing results, the MG-RAST output was filtered to only include bacterial and archaeal taxa. MG-RAST complete functional annotations of both the metagenomic and metatranscriptomic data were used for the assembly-free analysis of the community function.

Apart from these assembly-free community analyses, any overlapping paired-end reads from SD3 were joined with PANDASeq^39^ (with threshold parameter *t*=0.9) before the removal of potential PCR duplicates using custom scripts (available upon request). Read clipping, quality trimming and filtering of sequence reads was performed with the trim-fastq.pl script from the PoPoolation suite^40^. Four base pairs were hard-clipped from the 5' of all raw reads, and reads were filtered at or above a mean QV of 30 , and a minimum length of 40 bp. The quality of the resulting reads and the presence of remaining adaptor sequence contamination were assessed using FastQC (http://www.bioinformatics.babraham.ac.uk/projects/fastqc/).

To reduce the sample complexity and to improve the efficiency of the assembly process, quality-filtered, combined metagenomic and metatranscriptomic reads were initially mapped against the draft genome sequence of *Candidatus* Microthrix parvicella Bio17-1 (ref. 17). Mapped reads were extracted from the pool of reads and assembled separately with IDBA-UD^41^ (v.1.1.10), with the following parameters: --pre_correction --mink 35 --maxk 75 --step 2 --num_threads 12 --similar 0.97 --min_count 3. The remaining unmapped reads were binned as pairs according to low and high G+C percentage content, with an inclusive cut-off value of 50% G+C, and assembled as above. This strategy resulted in the generation of 1,739,837 additional base pairs of assembled sequence data compared with a direct assembly. Assemblies were merged using minimus and scaffolded using Bambus2 (AMOS tool suite^42^).

Assembled contigs longer than 500 bp, representing the first set, were grouped by a reference-free binning algorithm that has recently been developed by some of the authors^16^. The algorithm first computes the pentanucleotide frequency of each contig, which then allows representation of each contig as a point in a 512-dimensional Euclidean space (512 is the number of unique pentanucleotides after taking reverse complements into account). After a centred log-ratio transformation on each point^43^, the sets of points were used as input for the Barnes–Hut Stochastic Neighbourhood Embedding (BH-SNE) algorithm^44^, which produced a two-dimensional map (embedding) of the original signatures (Fig. 2a). Binning of points was then carried out using the Expectation–Maximisation (EM) algorithm on a postulated two-dimensional Gaussian Mixture model^45^, where the means of the Gaussian components of interest were initialized by the user and the covariance matrices were initialized by diagonal matrices with small positive entries. For the results reported in this work, we initialized EM with one Gaussian component per expected cluster following visual inspection. Contigs from the resulting clusters were extracted as contig groups and used as reference sequences to recruit sequence reads from the original, quality-filtered data set. Non-mapped paired-end reads were extracted and merged. A second iterative round of assembly was performed on each set of recruited reads separately and BH-SNE profiling was conducted as described above (except that this time a minimal contig size of 1,000 bp was used). Contig groups resulting from the second BH-SNE iteration were used once more for read recruitment. G+C percentage was calculated per base using in-house scripts (available upon request).

For coverage and gene expression analyses (SD3, SD5, SD6 and SD7), metagenomic and metatranscriptomic reads were mapped onto reconstructed genomic fragments from SD3 using Bowtie2 (ref. 46) (using ‘very sensitive-local’ parameters) and BWA^47^ (using default parameters except for the –M option). Gene expression levels were determined using Cufflinks^48^ on the basis of the BWA read mappings.

Metagenomic FPKM^48^ (fragments per 1 kb of sequence per 10^6^ mapped reads) and coverage values corresponding to each predicted gene in each of the CGs were obtained for the different time points (Supplementary Data 2 and Supplementary Data 3).

To estimate relative population sizes within the community, we devised a measure analogous to the RPKM^49^ (reads per 1 kb of sequence per 10^6^ mapped reads) measure, widely used for reporting the normalized abundance of, for example, transcripts and we defined this as follows:


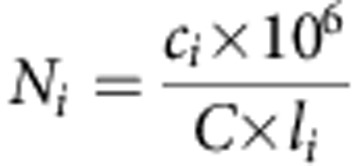


where *N*_*i*_ is the relative size of the population corresponding to CG*i*; *c*_*i*_ is the number of reads mapped to CG*i*; *C* is the total number of metagenomic reads mappable to all of the CGs; and *l*_*i*_ is the length of CG*i* in bp.

To account for the differences in observed expression levels linked to differing population sizes and to allow comparative analyses between different CGs as well as the different time points, genes were only considered to be expressed per CG per time point, if their metatranscriptomic FPKM values were ≥50 × *N*_*i*_ (Supplementary Data 2 and Supplementary Data 3).

### Metaproteome processing and analysis

Five microlitres of the protein extract obtained from SD3, SD5, SD6 and SD7 as previously described^12^,^13^ were mixed with 1.25 μl of XT sample buffer and 0.25 μl of XT reducing agent (Bio-Rad). After 10 min of denaturation at 70 °C, 5 μl the sample was subsequently separated by 1D SDS–PAGE (Criterion precast 1D gel, Bio-Rad). The gel was then stained with Imperial stain (Coomassie-Blue R250, Thermo Fisher Scientific) and cut into uniform 2 mm bands^50^. After in-gel reduction and alkylation, tryptic digestion was performed. Resulting peptides were separated by liquid chromatography (LC) using an Easy-nLC column (Proxeon, Thermo Fisher Scientific). Separation was performed using a 75 μm ID fused silica column packed with 20 cm of ReproSil Pur C18-AQ 3 μm beads (Dr Maisch). Before column separation, the samples were loaded onto a fritted 100 μm ID fused silica trap packed with 2 cm of the same material. The peptide mixture was separated using a binary solvent gradient to elute the peptides. Solvent A was 0.1% formic acid in water. Solvent B was 0.1% formic acid in acetonitrile. The peptide fractions were pooled in consecutive pairs, concentrated and resuspended up to 20 μl in solvent A. Eight microlitres of each pooled sample was injected per LC analysis. The three-step elution programme was operated at a flow rate of 0.3 ml min^−1^ consisting of (1) a gradient from 2 to 35% solvent B over 60 min, (2) a 10-min wash at 80% solvent B and (3) a 20-min column re-equilibration step at 2% solvent B.

Mass spectra were acquired on an LTQ-Orbitrap Elite (Thermo Fisher Scientific). The instrument was operated on an 11-scan cycle consisting of a single Fourier transformed (FT) precursor scan at 30,000 resolution followed by 10 data-dependent MS/MS scan events using higher-energy collisional dissociation at 15,000 resolution in the FT Orbitrap. The precursor scans had a mass range of 300–2,000 *m/z*, and an automatic gain control setting of 10^6^ ions. The MS/MS scans were performed using a normalized collision energy of 35 and an isolation width of 2 *m/z*. The data-dependent settings included monoisotopic precursor selection and charge state filtering that excluded unassigned and single charge states. Dynamic exclusion was enabled with a repeat count of 1, a repeat duration of 10 s, an exclusion list size of 500 and an exclusion duration of 180 s. Exclusion mass width was ±5 p.p.m. relative to mass.

LC-MS/MS analysis resulted in the generation of 271,915 mass spectra for SD3, 118,386 mass spectra for SD5, 102,916 mass spectra for SD6 and 105,328 mass spectra for SD7.

### Composite genome and expression analyses

*Gene calling and annotation*. The assembled CGs were submitted for gene calling and annotation to RAST^51^ with default parameters except for Domain (Bacteria), Genetic code (11), Sequencing method (other), FIGfam version (release 63) and with the Build metabolic model option selected.

*Taxonomic affiliation*. The taxonomies of the reconstructed CGs were determined using the AmphoraNet^24^ webserver. A taxon name was assigned when at least 75% of the identified marker genes resulted in a concordant taxonomy, and a putative taxon name was assigned when at least 50% of the identified markers resulted in a concordant taxonomy.

*Completeness and composition of composite genomes*. Genome completeness of the reconstructed CGs was estimated on the basis of 40 universal single copy genes^52^. For this, the functional annotation of the predicted proteins in each CG was obtained using the WebMGA server^53^ using the ‘cog’ analysis option. Functional compositions of the CGs and of their expressed genes were obtained from COG category counts, which were normalized by the total number of predicted features per CG.

*Comparative analysis of Candidatus Microthrix parvicella-like sequences*. Draft genome sequences for *Candidatus* Microthrix parvicella strains Bio17-1 (ref. 17) and RN1 (ref. 18) were obtained from the GenBank database (Assembly ID GCA_000299415.1 and GCA_000455525.1, respectively). Sets of orthologous genes were built using RAST’s ‘sequence based comparison’ tool.

*Variant identification*. SNPs were identified by separately mapping metagenomic and metatranscriptomic reads against the reconstructed CG assemblies using Bowtie2 and BWA (as described above). SNPs were identified from each of the mappings using mpileup (SAMtools^54^), the UnifiedGenotyper (Genome Analysis Tool Kit^55^) and Freebayes^56^. The intersection of identified SNPs from all the aforementioned methods was obtained using the vcf-isec utility from the VCFtools suite and was considered for subsequent analyses. SD3 variant amino-acid sequences were included in the amino-acid sequence databases generated on the basis of called and annotated genes. This database was used for subsequent protein identification on the basis of the generated metaproteomic data (see below). Variant frequencies were separately estimated from mapped metagenomic and metatranscriptomic reads. Only variants in regions with a minimum read depth (coverage) of 10 for both metagenomic and metatranscriptomic data were considered. Variant density per CG population was calculated by normalizing the SNP density (number of SNPs per kb) by the relative population size, which in turn was inferred from the fraction of metagenomic sequencing reads mapped onto the individual genomic reconstructions.

*Protein identification*. MS/MS spectra were searched against the generated amino-acid sequence database (containing the predicted proteins including all variants of the 10 reconstructed CGs and common contaminants) using the X!Tandem algorithm^57^. The resulting peptide identifications were validated using the Trans-Proteomic Pipeline^58^. The X!Tandem parameters included precursor and fragment ion mass tolerances of 15 p.p.m., a static modification of 57.021464 Da on cysteine residues and a potential modification mass of 15.994915 Da on methionine residues. The search allowed for semi-tryptic cleavages up to two missed cleavages. The database search results were validated and proteins were inferred at ~1% false discovery rate using the PeptideProphet, ProteinProphet and iProphet tools from the Trans-Proteomic Pipeline software suite^58^,^59^,^60^.

*Protein quantification*. Relative protein quantification was performed using the normalized spectral index (NSI) measure using an in-house software tool called NSICalc (details available upon request). The tool was adapted from the method by Griffin *et al*.^61^ Briefly, the NSI combines peptide count, spectral count and MS/MS fragment ion intensity for quantification and normalizes these values by the length of each protein. This strategy incorporates measurable peptide intensities while removing some of the biases of using spectral counts when comparing large and small proteins. NSI values were log_2_ normalized before comparison across proteins to obtain relative quantification ratios.

Metaproteomic analyses led to peptide matching against the amino-acid database of 43,214 spectra, which in turn provided abundance data on a total of 1,815 proteins for SD3.

*Analysis of the long-chain fatty acid-CoA ligases of CG8b*. Amino-acid sequences of genes annotated as long-chain-fatty-acid-CoA ligases by RAST from CG8b were aligned using Expresso^62^ using default parameters. Sequence similarities were determined using the SIAS server (http://imed.med.ucm.es/Tools/sias.html). A dendrogram based on pairwise comparisons of amino-acid sequence similarities was obtained using the hierarchical clustering function in R. Abundance values were extracted from the mapped metagenomic, metatranscriptomic and metaproteomic data.

## Author contributions

E.E.L.M., H.R., L.A.L. and P.W. sampled the treatment plant and extracted biomolecules; E.E.L.M., N.P., C.C.L., S.N., J.L., P.M., N.D.H., A.H.-B., L.W., C.M.L., N.V. and P.W. analysed the (meta)genomic and/or metatranscriptomic data; E.E.L.M., N.P., M.R.H. and R.L.M. analysed the metaproteomic data; E.E.L.M., N.P. and C.G. analysed the metabolomic data; E.E.L.M, N.P. and P.W. designed the study and wrote the manuscript. All the authors discussed the results and commented on the manuscript.

## Additional information

**How to cite this article:** Muller, E. E. L. *et al*. Community-integrated omics links dominance of a microbial generalist to fine-tuned resource usage. *Nat. Commun.* 5:5603 doi: 10.1038/ncomms6603 (2014).

**Accession codes:** All raw sequence data have been deposited in the NCBI Bioproject with the accession code PRJNA230567. MG-RAST-based analysis results of the metagenomic and metatranscriptomic data can be found at http://metagenomics.anl.gov/linkin.cgi?metagenome=4566023.3 and http://metagenomics.anl.gov/linkin.cgi?metagenome=4566620.3, respectively. RAST IDs from 6666666.37195 to 6666666.37203 correspond to CG1 to CG9 assemblies and annotations are available through the RAST guest account at http://rast.nmpdr.org, using ‘guest’ as login as well as password. All proteomic data has been deposited in the PeptideAtlas mass spectrometry raw file repository at http://www.peptideatlas.org/ under the ‘datasetIdentifier’ PASS00359, PASS00576, PASS00577 and PASS00578 for SD3, SD5, SD6 and SD7, respectively.

## Supplementary Material

Supplementary Figures Supplementary Figures 1-10

Supplementary Data 1 Characteristics of the reconstructed composite genomes at SD3.

Supplementary Data 2 List of genes for each composite genome (CG) with corresponding transcript levels and protein abundances for the sample obtained on SD3.

Supplementary Data 3 List of genes for each composite genome (CG) with corresponding transcript levels and protein abundances for samples obtained on SD5 to SD7.

Supplementary Data 4 List of single nucleotide polymorphisms (SNPs) detected for each composite genome (CG) for samples obtained on SD3, SD5, SD6 and SD7.

## Figures and Tables

**Table 1:** The characteristics of composite genomes CG5 and CG8b at different sampling time points.

	**SD3**		**SD5**		**SD6**		**SD7**	
	**CG5**	**C8b**	**CG5**	**CG8b**	**CG5**	**CG8b**	**CG5**	**CG8b**
Average composite genome coverage ( × )	9.65	23.36	30.02	45.74	20.54	65.57	35.06	81.81
Proportion of total metagenomic reads mapped per composite genome (%)	8.10	36.50	16.81	37.48	11.27	51.38	13.71	47.85
Percentage of ORFs expressed at the RNA level	92.7	45.8	78.9	25.1	85.3	32.0	87.0	36.8
Number of detected variants (based on the metagenomic data)	5,428	11,702	42,250	11,588	29,431	12,596	37,699	11,517
Number of detected variants (based on the metatranscriptomic data)	11,481	777	24,353	1,366	26,227	2,923	28,247	3,504
Variant density per CG population	2.34E−04	7.43E−05	8.78E−04	7.17E−05	9.13E−04	5.68E−05	9.61E−04	5.58E−05
