# Supplementary material for: Community-integrated omics links dominance of a microbial generalist to fine-tuned resource usage
Source: Nat Commun. 2014 Nov 26;5:5603. doi: 10.1038/ncomms6603 (PMC4263124; doi:10.1038/ncomms6603)
Supplement: Supplementary Figures — 1-10 [file ncomms6603-s1.pdf]

Supplementary information

Supplementary Figures

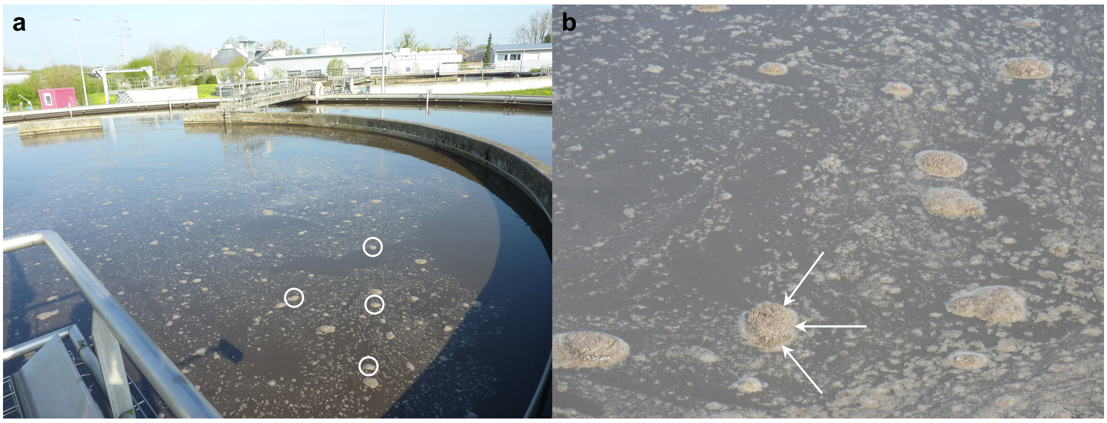

Supplementary Figure 1 | Photographs of OMMCs located at the surface of the anoxic tank of the municipal wastewater treatment plant at Schifflange (Luxembourg). (**a**) Individual islets (biological replicates) are highlighted by white circles. (**b**) Detailed view of distinct islets. The delineation of an exemplary islet is indicated by arrows.

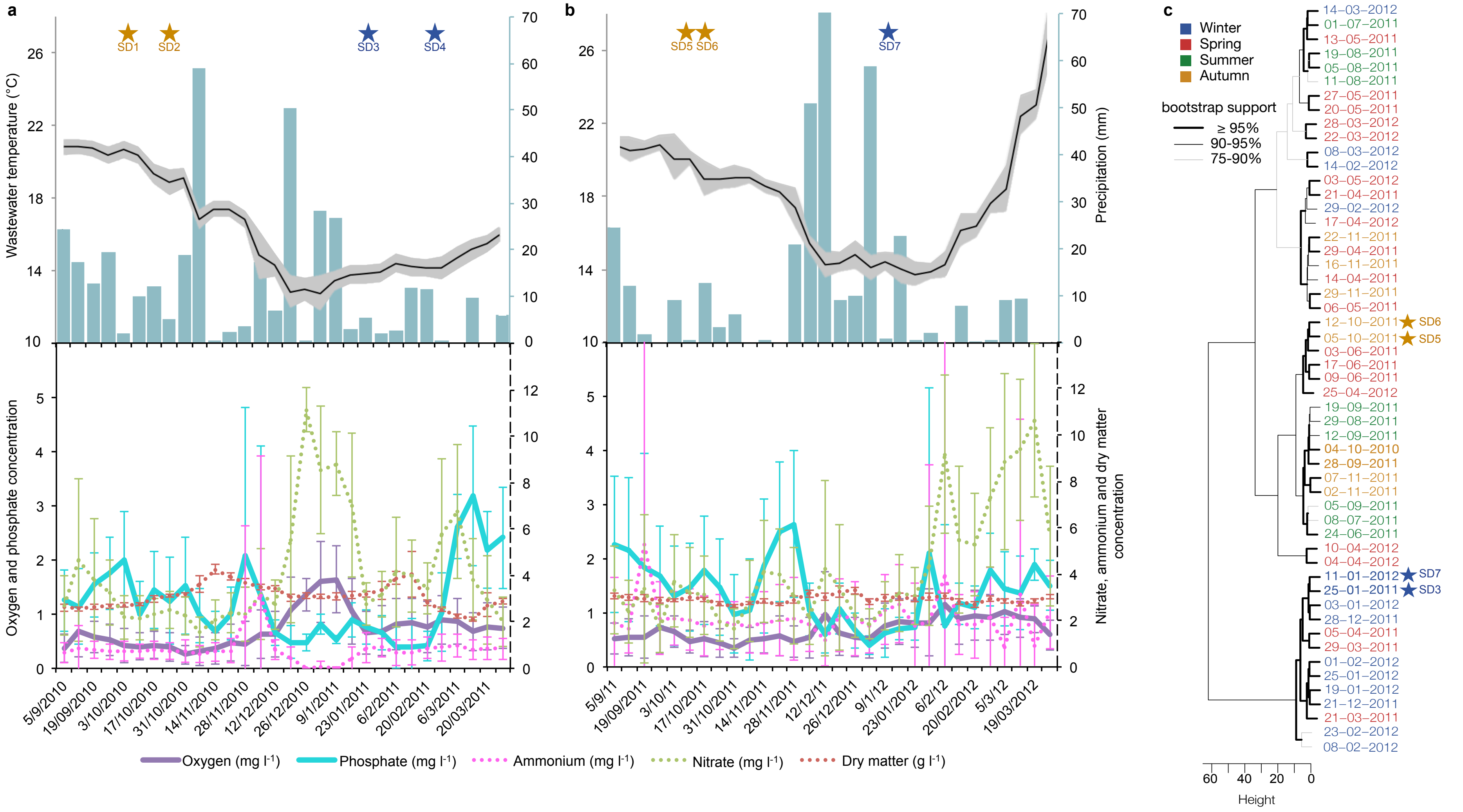

Supplementary Figure 2 | Environmental fluctuations within the sampled biological wastewater treatment plant. (**a**) and (**b**) Weekly average temperature of the influent wastewater (measured each second hour; black line) with the standard deviation indicated in the grey area and weekly precipitation shown by blue bars (upper graphs) as well as weekly averages of five physico-chemical parameters measured each

second hour (lower graphs). (**a**) Data for the period of the 5 September 2010 to the 27 March 2011. (**b**) Data for the period of the 5 September 2011 to the 27 March 2012. (**a**) and (**b**) Stars indicate the sampling dates for which samples were obtained for multi-omic analyses (SD1: 4 October 2010; SD2: 25 October 2010; SD3: 25 January 2011; SD4: 23 February 2011; SD5: 5 October 2011; SD6: 12 October 2011 and SD7: 11 January 2012). (**c**) Hierarchical clustering of the OMMC samples based on 7 physico-chemical parameters (averages of the measurements taken each second hour for the 3 days prior to sampling) including wastewater influent temperature, dissolved oxygen, phosphate, ammonium, nitrate, dry matter and pH. The stars denote sampling timepoints from which samples were used for the integrated omic analyses.

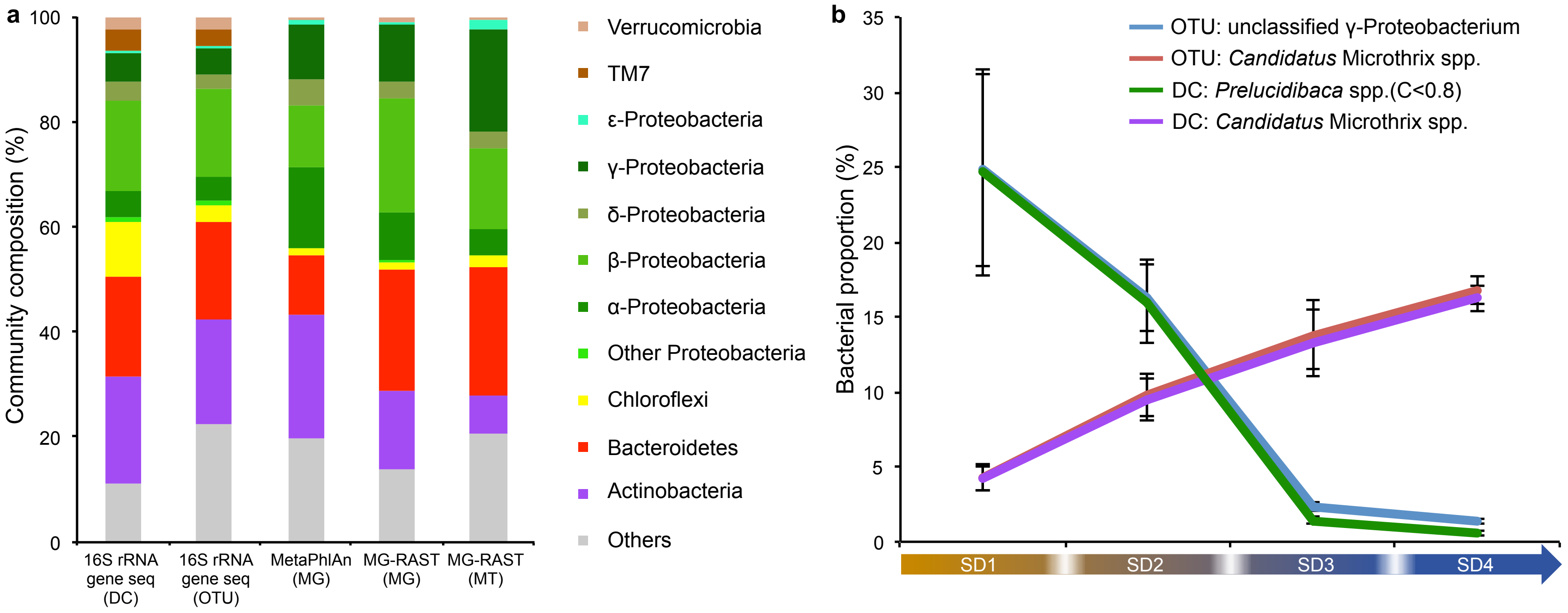

Supplementary Figure 3 | Microbial community profiles. (**a**) Comparison of the average genus-level abundances of the two dominant populations when OTU clustering or direct classification (DC) were applied. The most abundant microbial population in the winter (SD3 and SD4) was identified as *Candidatus* Microthrix spp. using both strategies, whereas the dominant population in autumn (SD1 and SD2) was tentatively identified (confidence level below 0.8) as *Perlucidibaca* spp. using DC or as an unclassified γ-Proteobacterium using OTU clustering. (**b**) Fractions of taxa identified across the consortia sampled on SD3 sample I using different microbial community profiling approaches.

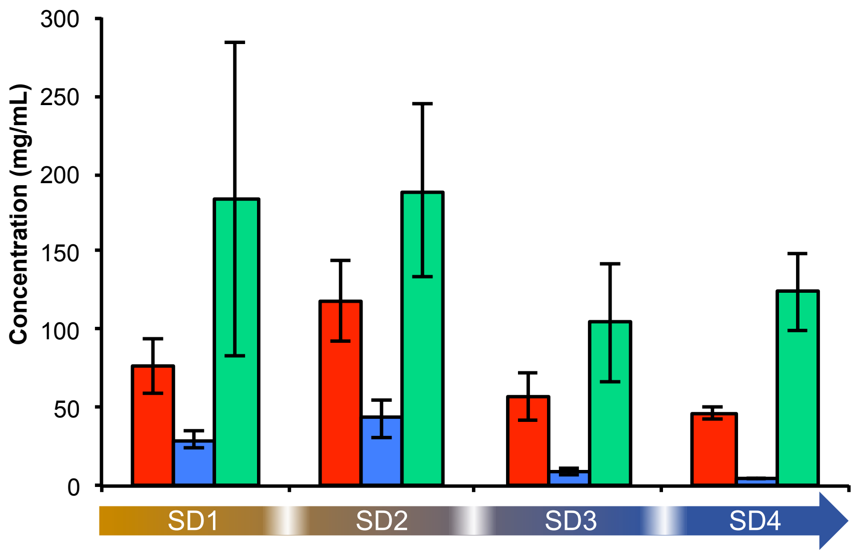

Supplementary Figure 4 | Organic substrates available to the OMMCs on the four distinct sampling dates. Concentrations of total protein (red bars), major fatty acids (blue bars) and total carbohydrates (green bars) in the wastewater on the four sampling dates are indicated. The values shown are the mean ± s.d. (*n*=15).

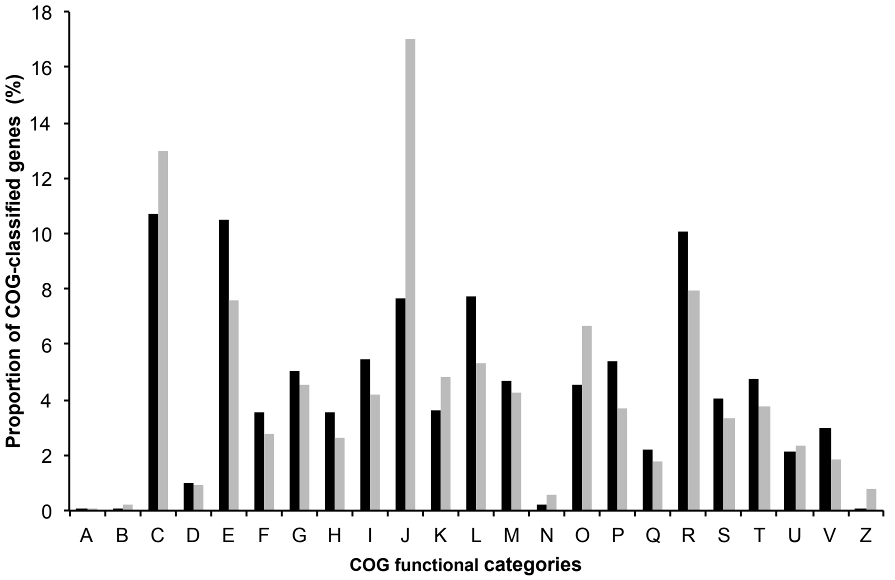

Supplementary Figure 5 | Functional microbial community profile derived from assembly-free analyses of metagenomic and metatranscriptomic data. The proportion of genes for each COG category as predicited by MG-RAST at the metagenomic (black; MG-RAST ID 4566023.3) and metatranscriptomic (grey; MG-RAST ID 4566620.3) levels are indicated. The individual COG categories are: A, RNA processing and modification; B, Chromatin structure and dynamics; C, Energy production and conversion; D, Cell cycle control, cell division, chromosome partitioning; E, Amino acid transport and metabolism; F, Nucleotide transport and metabolism; G, Carbohydrate transport and metabolism; H, Coenzyme transport and metabolism; I, Lipid transport and metabolism; J, Translation, ribosomal structure and biogenesis; K, Transcription; L, Replication, recombination and repair; M, Cell wall/membrane/envelope biogenesis; N, Cell motility; O, Posttranslational modification, protein turnover, chaperones; P, Inorganic ion transport and metabolism; Q, Secondary metabolites biosynthesis, transport and catabolism; R, General function prediction only; S, Function unknown; T, Signal transduction mechanism; U, Intracellular trafficking, secretion and vesicular transport; V, Defence mechanisms; Z, Cytoskeleton.

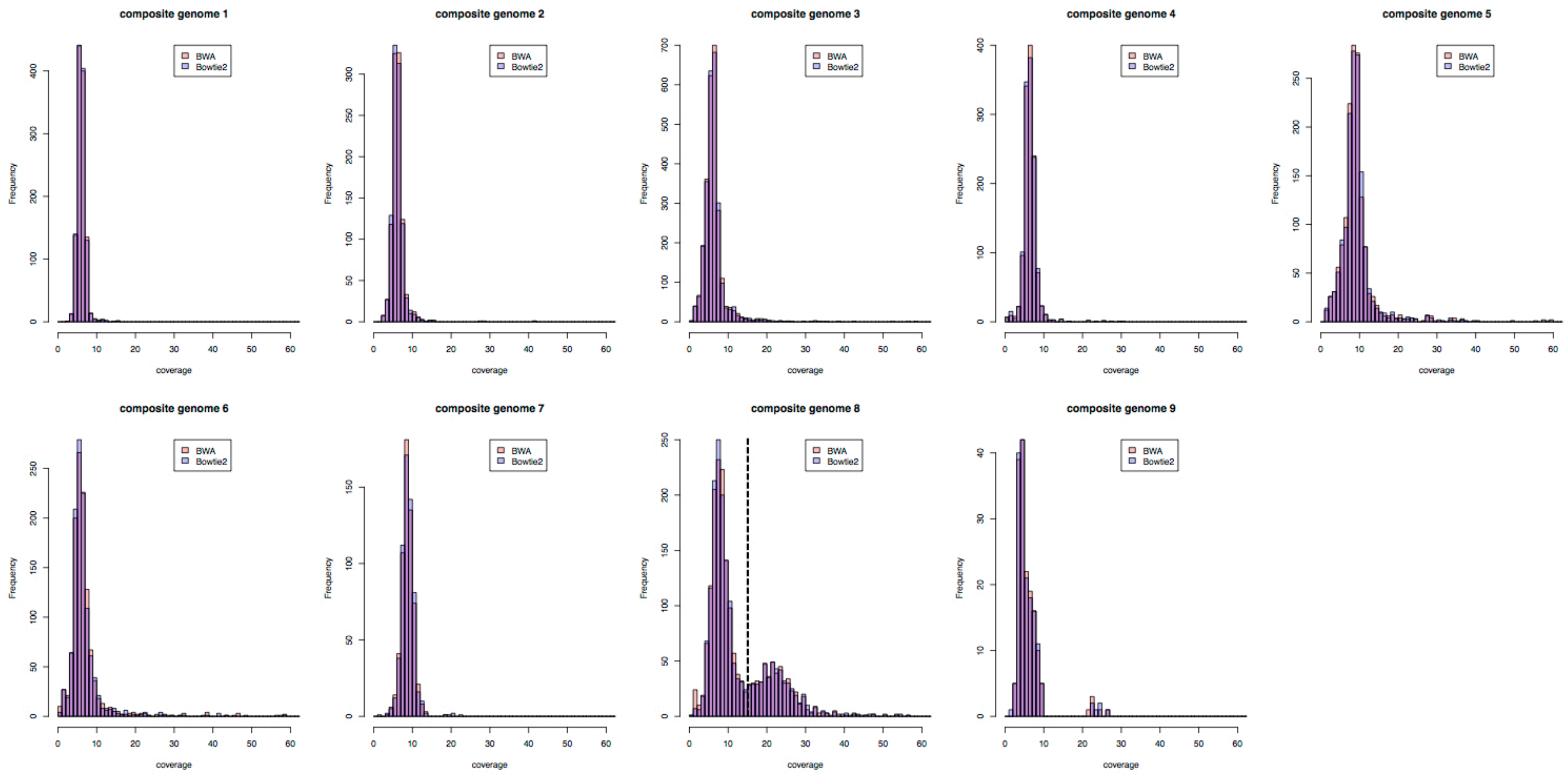

Supplementary Figure 6 | Metagenomic read coverages for individual composite genome (CG) groups. Distribution of the average coverage values for each CG group obtained by mapping the metagenomic read data back to the assembled CG contigs using two different read mapping algorithms (BWA and bowtie2). For CG8, the dashed line indicates the coverage value, i.e. 15 X, that allowed separation of the two subpopulations, CG8a and CG8b.

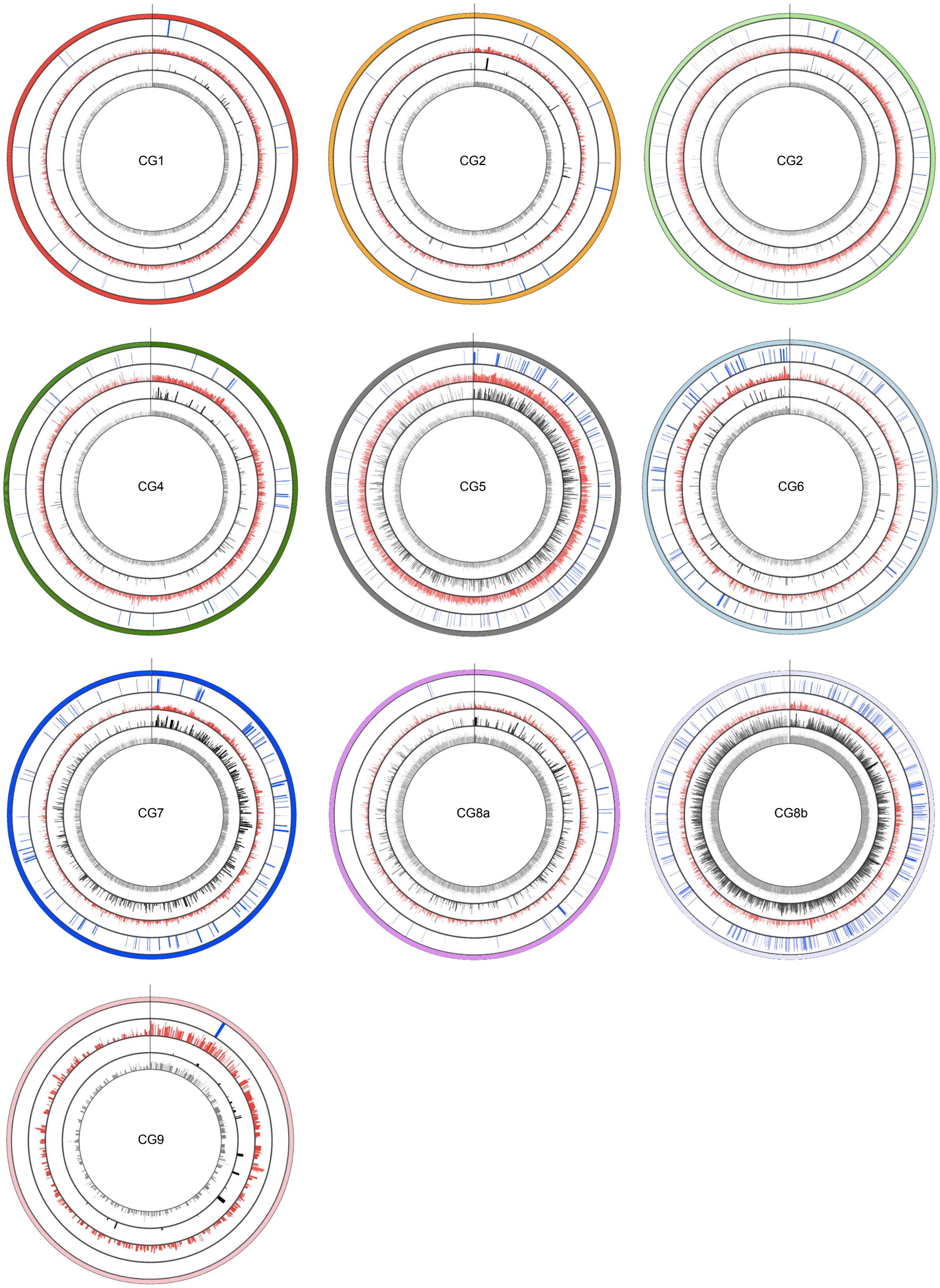

Supplementary Figure 7 | Genome-wide gene expression levels for the ten reconstructed composite genomes (CGs). Tracks (from the innermost concentric track to the outermost): $\log_{10}$ of the metagenomic fragments per 1 kb of sequence per

10^6 mapped reads (FPKM) (dark grey), log_{10} of the number of detected SNP per gene (black), log_{10} of the metatranscriptomic FPKM (red), log_2 of the protein expression levels in Normalised Spectral Indices (blue), and reconstructed contigs ordered by size. Track scales are identical across plots.

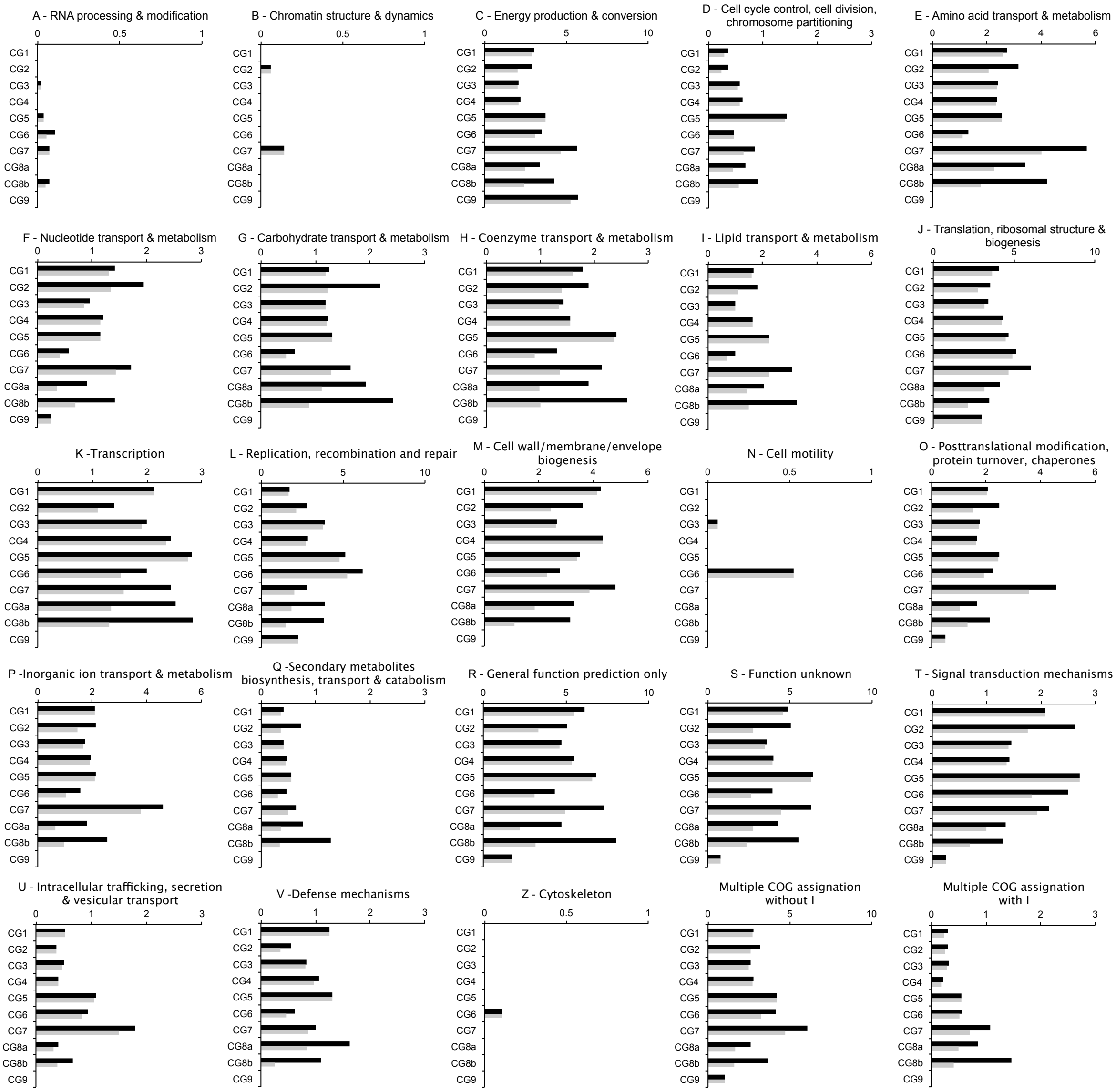

Supplementary Figure 8 | Gene expression within different functional gene categories (COGs). Relative proportions of the functional gene categories encoded by the different composite genomes and corresponding transcript levels displayed by category (black bars indicate representation at the genomic level, grey bars at the transcriptomic level).

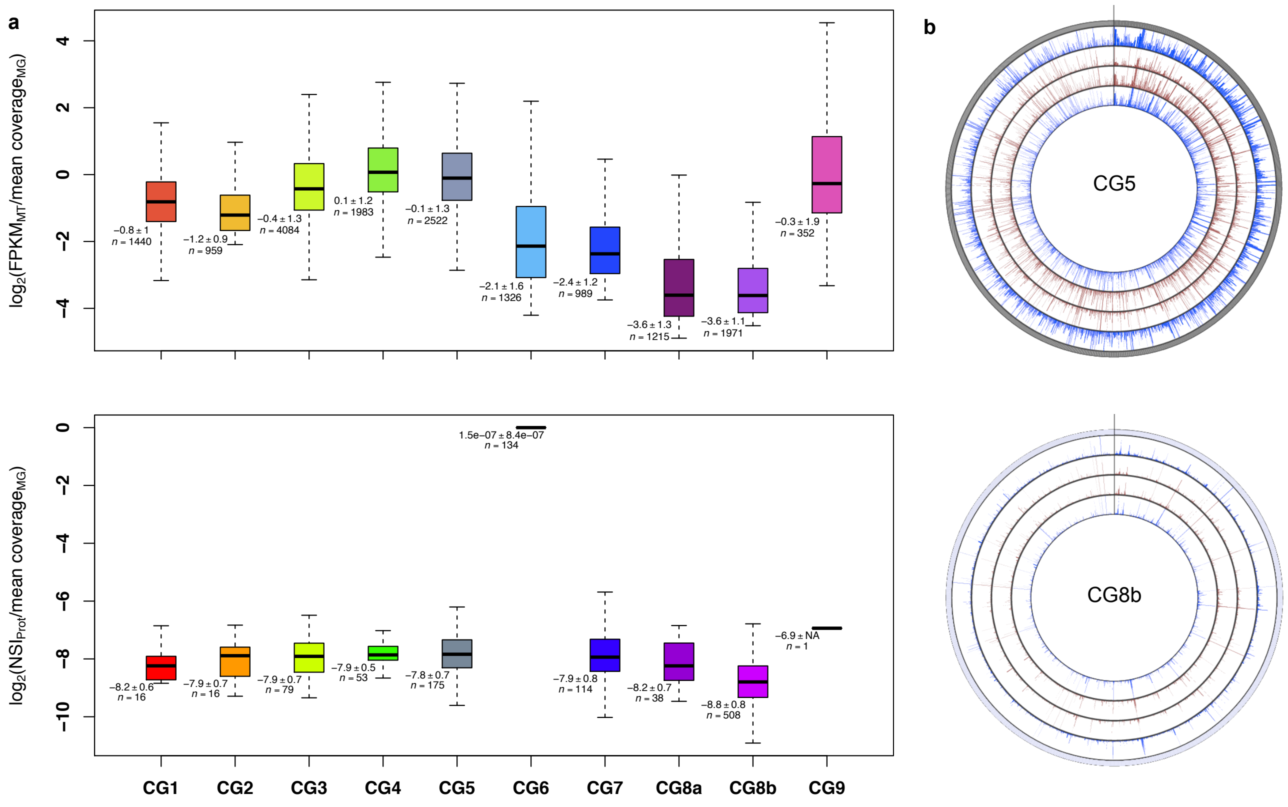

Supplementary Figure 9 | Population-level gene expression. (a) RNA expression levels represented as log_2 ratios of the metatranscriptomic fragments per 1 kb of sequence per 10^6 mapped reads (FPKM) values and average metagenomic coverage per locus (upper plot), and protein expression levels represented as log_2 ratios of the Normalised Spectral Index (NSI) and average metagenomic coverage per locus (lower plot).

The values shown next to each box are median ± s.d. and *n* is the number of features. Boxplots represent the lower quartile, median and upper quartile. Whiskers are placed at 1.5× interquartile range beyond the lower and upper quartiles. (**b**) Genome-wide transcript expression levels of CG5 and CG8b on four different sampling dates. Tracks represent metatranscriptomic fragments per 1 kb of sequence per 10^6 mapped reads (FPKM) normalised according to population size (Methods). From the outermost concentric track to innermost: SD3, SD5, SD6 and SD7.

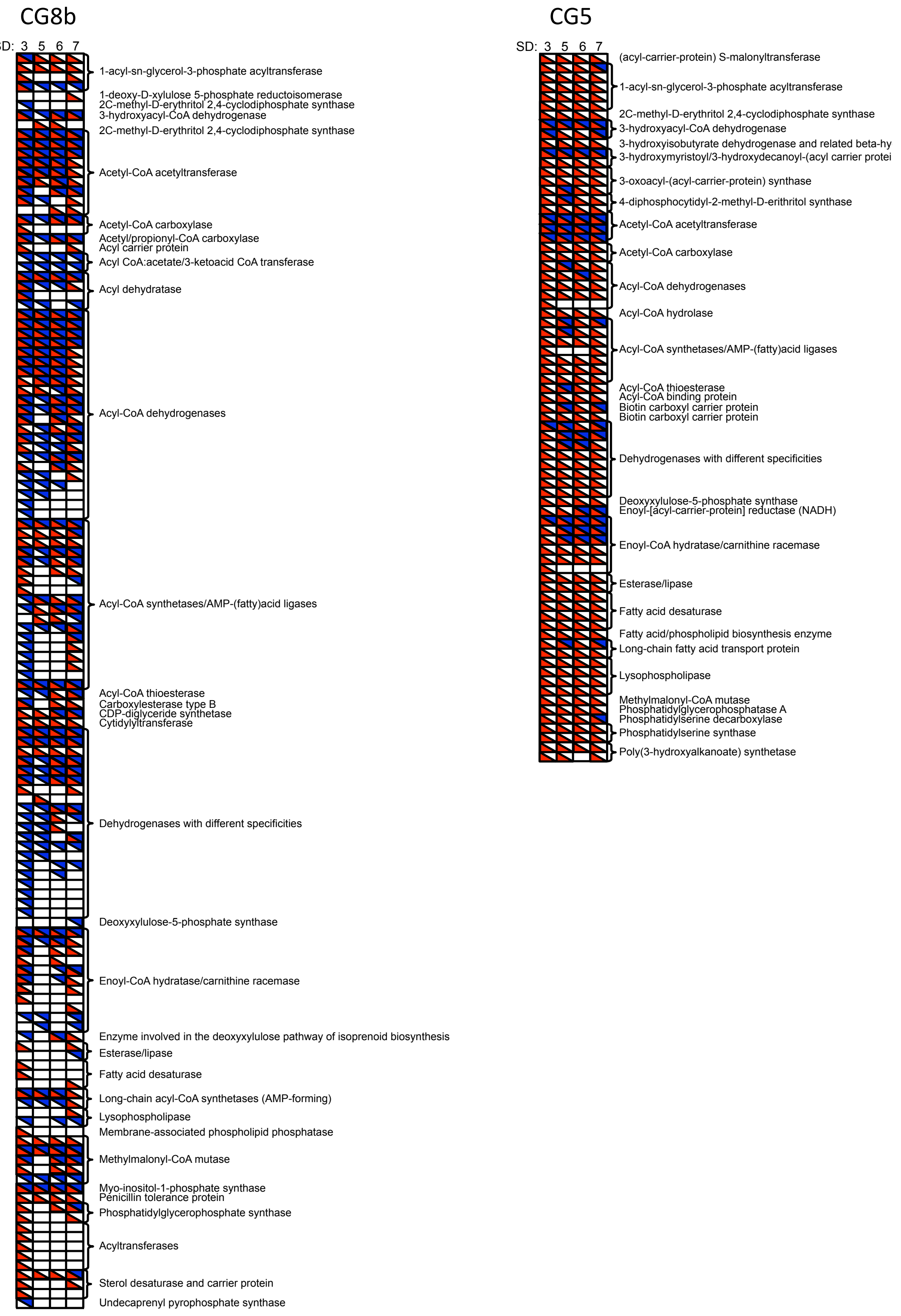

Supplementary Figure 10 | Patterns of gene expression for lipid transport and metabolism by *Candidatus* Microthrix parvicella and the CG5 population. Qualitative gene expression patterns of genes belonging to the COG category "I - lipid

transport and processing” encoded by *Candidatus* Microthrix parvicella (CG8b) and the CG5 population at the metatranscriptomic (red) and metaproteomic (blue) levels across four sampling timepoints.
